# Odontogenic Keratocyst of the Maxilla With Fungal Sinusitis: A Rare Case

**DOI:** 10.7759/cureus.67038

**Published:** 2024-08-16

**Authors:** Amrinder Singh, Rishabh Kasrija, Ajay Mittal, Ankush Gupta, Harmandeep Kaur

**Affiliations:** 1 Department of Oral and Maxillofacial Surgery, Guru Nanak Dev Dental College & Research Institute, Sunam, IND; 2 Department of Oral and Maxillofacial Surgery, JSS Dental College, Mysuru, IND

**Keywords:** sinusitis, maxilla, odontogenic cyst, fungal infection, enucleation

## Abstract

Odontogenic keratocysts (OKCs) in the maxilla are rare. Although destructive, their clinical presentation can mimic inflammatory conditions like radicular cysts and osteomyelitis. OKCs originate primarily from odontogenic sources. On radiography, they present a unilocular to multilocular radiolucency and may involve the maxillary sinus. Enucleation with chemical cauterization is the best treatment for limiting cyst recurrence. However, superaided infections, such as fungal sinusitis, can adversely affect the condition and require a more comprehensive treatment plan. The present case report describes an OKC of the upper jaw involving the maxillary sinus in a 42-year-old male with a superadded fungal infection. The treatment plan included enucleation with chemical cauterization along with inferior meatal antrostomy. In addition, an antifungal protocol was instituted. A follow-up period of one year was not associated with any complications.

## Introduction

An odontogenic keratocyst (OKC), also described as a keratocystic odontogenic tumor (KCOT), is a distinctive and aggressive form of odontogenic cyst that is sourced from the dental lamina and its remnants [1]. Due to their high propensity of recurrence and a tendency to infiltrate neighboring tissues, OKCs demonstrate a behavior more akin to that of benign neoplasms than conventional cysts. Despite the potential for significant morbidity associated with these lesions, the World Health Organization (WHO) has been subject to uncertainty. Initially labeled KCOTs in 2005, the WHO later reverted to OKCs in 2017, citing a lack of substantial evidence to support their classification as tumors [2,3].

OKCs are primarily observed in the mandible, especially in the molar-ramus region, representing approximately 70-80% of cases [4]. The mandible is implicated with a ratio of 2:1 to the maxilla, demonstrating a pronounced propensity for the involvement of the mandibular ramus, whereas the maxilla is involved in merely 13%. Maxillary sinus OKCs are remarkably uncommon, and the posterior maxilla includes 23% of all OKCs and constitutes less than 1% of all documented cases, with an even smaller proportion occurring in the anterior maxilla [5]. The aggressive nature of OKCs is emphasized by their tendency to recur, which can reach up to 60% following conservative treatment [6]. This recurrence is linked to the thin, delicate lining of the cyst and potential satellite cysts within the bone [7].

Radiographically, OKCs commonly manifest as clearly delineated radiolucent lesions, appearing either unilocular or multilocular and frequently displaying scalloped or smooth borders [2]. Histopathological examination revealed a distinctive lining composed of parakeratinized stratified squamous epithelium, typically eight to 10 layers in thickness, featuring a corrugated surface and a basal layer of hyperchromatic cuboidal cells arranged in a palisaded manner [8].

Management strategies for OKCs encompass a spectrum of interventions, ranging from conservative measures such as marsupialization and decompression to more invasive surgical procedures such as enucleation with peripheral ostectomy and resection [9]. The selection of an appropriate treatment modality is guided by various factors, including the lesion size, location, and overall health status of the patient. Complementary therapeutic approaches, such as the application of Carnoy's solution and cryotherapy, have been employed as strategies to reduce the likelihood of a recurrence [10]. Moreover, the presence of secondary infections, such as fungal infections, may add complexity to clinical progression, requiring the administration of antifungal treatment alongside surgical intervention [11,12]. This paper presents a rare case of an OKC located in the maxilla that involved the maxillary sinus and was complicated by a superadded fungal infection.

## Case presentation

A 42-year-old male was reported to the Department of Oral and Maxillofacial Surgery with primary complaints of pain and swelling in the left maxilla persisting for six months. The patient reported a history of mild, continuous, non-radiating pain, fullness, or pressure localized to the upper left posterior maxillary region. This pain was exacerbated by mastication and alleviated by nonsteroidal anti-inflammatory intervention. The patient was fully oriented to time, place, and person and had no medical history indicative of any underlying systemic disease.

**Figure 1:**
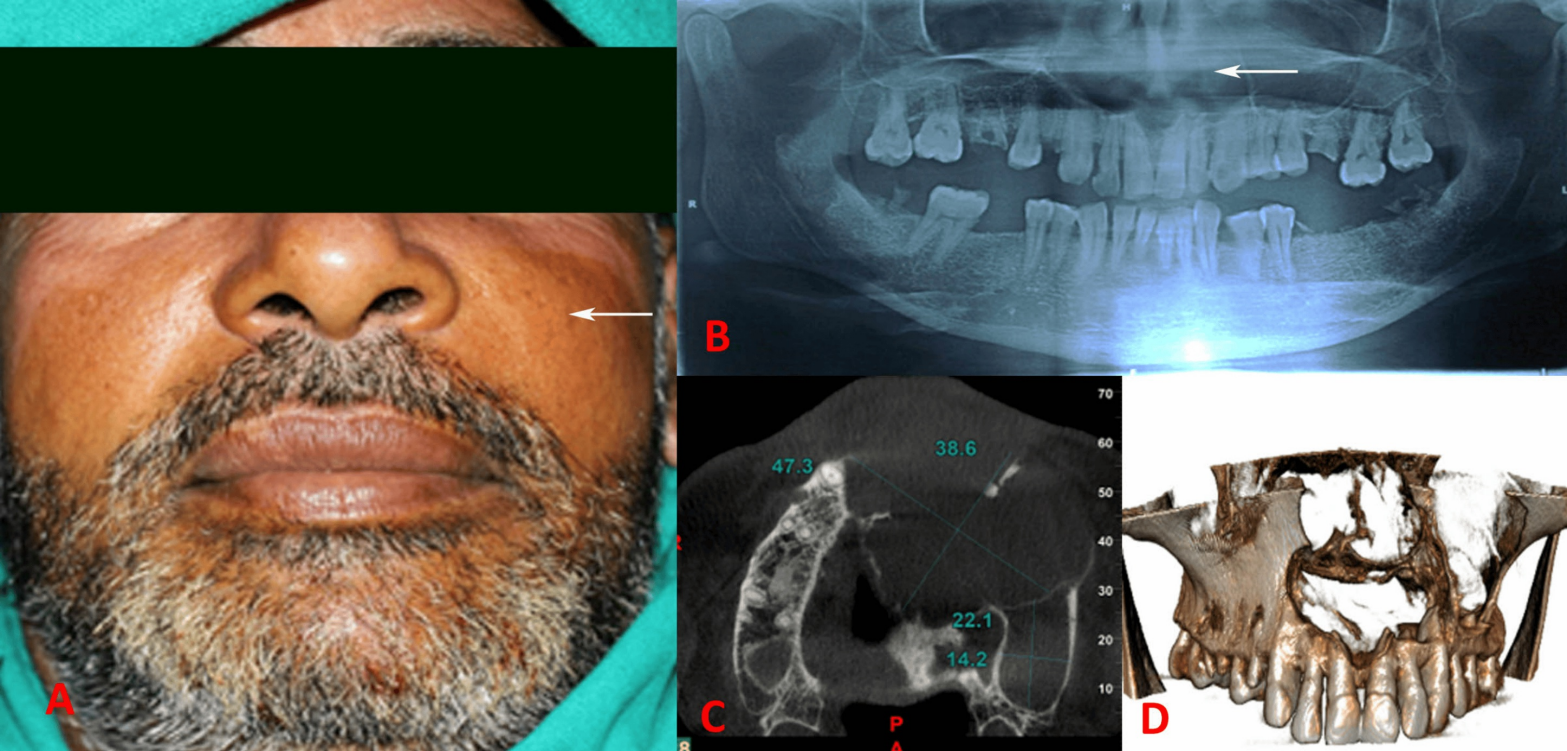
(A) Diffuse swelling on the left maxilla obliterating nasolabial fold, (B) OPG showing radiolucency extending from the left first molar to the anterior maxilla and involving the left maxillary sinus, (C) size of the cystic radiolucency, (D) CBCT showing extension of the cyst margins.

Extraoral examination revealed a diffuse swelling in the left maxillary region. This swelling extended mediolaterally from the left ala of the nose to the left zygomatic prominence and superoinferiorly from the infraorbital region to the upper lip, effectively obliterating the nasolabial fold (Figure 1A). Intraoral examination revealed a draining sinus in the left maxillary buccal vestibule, specifically in the maxillary second premolar region (tooth 24). The pus actively exuded from this sinus, characterized by a straw-colored yellow fluid with cheesy and thick contents. Pulp vitality test showed a nonvital nature of the right maxillary lateral incisor (12) to the left maxillary third molar (28) teeth.

An orthopantomogram (OPG) revealed a well-corticated radiolucency associated with the first molar tooth, with the radiolucent area extending toward the floor of the nasal fossa (Figure 1B). Cone-beam computed tomography (CBCT) demonstrated an ill-defined, large, expansile, multilocular osteolytic lesion extending from the maxillary right first premolar (tooth 14) to the maxillary left first molar (tooth 26). The lesion measured 47.3 mm x 38.6 mm in the anterior locule and 22.1 mm x 14.2 mm in the posterior locule (Figure 1C). Significant maxillary alveolar, palatal, and nasal bone destruction was observed. The lesion expanded superiorly into the nasal cavity, causing erosion of the floor and lateral wall of the nasal cavity and the nasal septum (Figure 1D). In addition, erosion of the mesial wall of the left maxillary sinus was observable, with the lesion protruding into the maxillary sinus.

**Figure 2:**
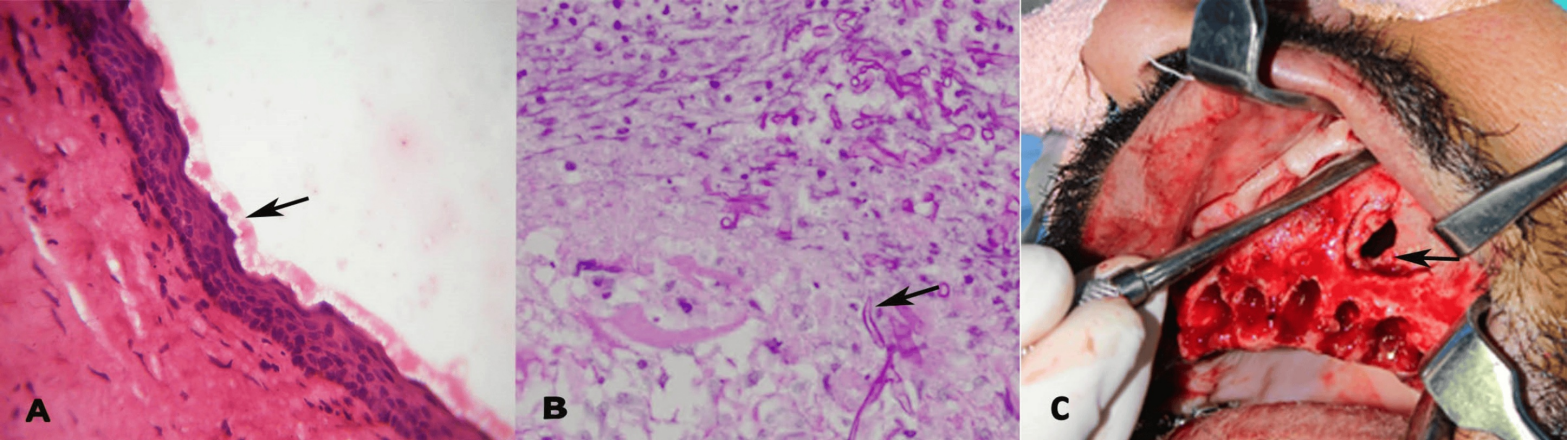
(A) Hematoxylin and eosin-stained section at 40X magnification showing the corrugated epithelial lining of the odontogenic keratocyst (OKC), (B) periodic acid Schiff-stained section at 40X magnification showing fungal hyphae in the connective tissue, (C) surgical enucleation of the cyst.

Following routine blood investigations to delineate diabetes, HIV positivity, and hematological disorders, an incisional biopsy was performed. Microscopic investigation revealed a uniform parakeratinized stratified squamous epithelium, eight to 10 cells thick, lining a thin fibrous connective tissue wall. The luminal surface exhibited a flattened parakeratotic epithelium with a corrugated appearance (Figure 2A). The basal layer consists of a palisaded arrangement of hyperchromatic cuboidal cells. The connective tissue stroma was edematous, showing diffuse lymphoplasmacytic cell infiltration. The fungal hyphae and spores were identified in the mucosal connective tissue using the periodic acid-Schiff stain, situated in close proximity to, yet not penetrating, the vessels, suggesting an infected OKC with superimposed deep fungal infection (candida) (Figure 2B).

Cyst enucleation was performed under general anesthesia. A crevicular incision was made, extending from the right maxillary first premolar (tooth 14) to the left maxillary third molar (tooth 28) region, followed by blunt dissection. The cystic lining was carefully enucleated along with the peripheral osteotomy, and the teeth from the right maxillary lateral incisor (tooth 12) to the left maxillary third molar (tooth 28) were extracted (Figure 2C).

The cavity was irrigated thoroughly with saline and 10% povidine-iodine. Chemical cauterization was performed using 5-fluorouracil. A gauze pack containing iodoform impregnated with anesthetic 2% lignocaine jelly was inserted into the body cavity, with one end protruding through the left nostril following an inferior meatal antrostomy. The flap was sutured using 3-0 silk sutures. The patient was instructed to take a regimen of amoxicillin 500 mg in conjunction with clavulanic acid 125 mg thrice daily for a period of five days. The initial antifungal regime of posaconazole 600 mg per day in two divided doses for three days, and a maintenance dose of 300 mg per day for two months was prescribed. The gauze pack was removed 24 hours later via the nostril. Follow-up visits were conducted seven days, two months, and one year postoperatively, with the patient showing no symptoms of sinusitis or any other complaints.

## Discussion

Maxillary sinus involvement in OKCs is rare, with most OKCs typically found in the posterior regions of the mandible or maxilla's posterior regions [13]. OKCs are frequently observed to form primarily within the alveolar ridge and remain restricted to osseous structures. As a cystic entity, the OKC typically enlarges by following the path of least resistance, which frequently entails propagation within the bone rather than perforating the floor of the sinus. However, there have been documented cases of OKCs invading the maxillary sinus, particularly in the middle turbinate [1,14]. These cases present challenges in diagnosis because of their symptomatic resemblance to other maxillary sinus lesions, such as sinusitis or polyps [15,16]. Maxillary sinusitis is characterized by extensive sinus opacification exhibiting indistinct margins and mucosal hypertrophy, in the absence of osseous destruction or involvement of dental structures. Sinus polyps present as soft tissue formations with smooth, poorly defined peripheries within the sinus cavity, leading to partial opacification while exerting no influence on dental elements or bone expansion [17]. In the current study, we report an unusual case of OKC with maxillary sinus involvement. The existing literature indicates that 23% of OKCs occur in the posterior maxilla and a minority, specifically less than 1%, of OKC cases manifest in the maxilla with sinus engagement [5,14].

Clinical features of OKCs in the anterior maxilla include a slight increase in volume, radiolucent images on radiographs, and hypodense, well-defined areas with disruption of the buccal cortex on computed tomography scans [2,15]. In the present case, the patient’s pressure and fullness indicated maxillary sinus involvement, even though the patient did not report any previous history of sinusitis. In rare cases, OKCs can invade unusual locations, such as the middle turbinate of the maxillary sinus, presenting as bony swelling with associated pain and swelling in the affected area. The obstruction of sinus ventilation and the development of anaerobic conditions due to blockage of the ostium by the OKC may have facilitated the germination of fungi specifically aspergillosis [16,18]. A previous study suggested that odontogenic infection is a risk factor for fungal sinusitis in the ipsilateral maxillary sinus [12,18]. The present case is a rarely reported incidence of super-aided fungal infection in OKC. It is crucial to acknowledge that OKCs may present a diagnostic challenge because of their potential resemblance to inflammatory lesions like radicular cysts and sinusitis [19]. This is primarily because individuals affected by this particular cystic neoplasm typically manifest inflammatory indicators, such as pain, swelling, and pus discharge. Comparable symptoms were evident in this case.

Radiologically, OKCs typically present as well-defined radiolucent areas, often with smooth and corticated borders [4]. These lesions can cause significant displacement of the teeth and resorption of the surrounding bone, making them easily distinguishable from other cystic formations [7,15]. In the maxilla, OKCs can extend into adjacent anatomical structures, including the nasal floor and maxillary sinus, as demonstrated in the present case with CBCT.

Although clinical and radiographic features may provide valuable insights, it is crucial to acknowledge that a definitive diagnosis can only be conclusively established through meticulous analysis of histopathological findings [8,16]. A noteworthy finding in our case was the presence of candidal elements, such as hyphae and spores, within the cystic lining and cystic epithelium extending into adjacent structures, such as the maxillary sinus. This secondary fungal infection, although rare, can complicate the clinical course and necessitate specific antifungal treatment alongside conventional surgical and medical management [13,18]. Given that our patient demonstrated immunocompetence, comprehensive excision of the cyst, along with sinus drainage and aeration facilitated through rhinoantrostomy, were deemed to be therapeutic measures for both OKC and fungal infection.

Enucleation, a frequently employed therapeutic approach for OKCs, encompasses total excision of the cystic epithelial lining to mitigate the risk of recurrence [9]. The incorporation of peripheral osteotomy additionally facilitates the complete eradication of any potentially infiltrative cells present at the periphery [5]. The removal of the affected teeth (right lateral incisor to left second molar in the present case), while considered drastic, is often indispensable in extensive cases to ensure thorough eradication of the lesion. The use of 5-fluorouracil for chemical cauterization is a notable method to treat the lining of cysts, aimed at reducing the recurrence rate [20], particularly in the present case, which is known for its recurrence potential [6,9]. Postoperative care with antibiotics, such as amoxicillin and clavulanic acid, helps prevent secondary infections, while follow-up visits are crucial for monitoring healing and early detection of recurrence. Inferior meatal antrostomy is particularly useful for better ventilation and drainage from the maxillary sinus in the management of fungal sinusitis or removal of cysts extending into the sinus cavity [18]. Studies have shown that OKCs have a higher recurrence rate than other odontogenic cysts, ranging from 7.1% to 45%, with an average rate of 22.5% [21]. A follow-up period of a minimum of two years is warranted.

## Conclusions

This report serves as a demonstration of the intricacies involved in the diagnosis and treatment of OKCs, particularly when further complicated by secondary fungal infections. The extensive destruction of the maxilla is a combined effect of fungal sinusitis and OKCs. Employing advanced imaging techniques, histopathological analysis, and a combination of surgical and medicinal interventions is essential for achieving favorable results. It is evident from the findings that a thorough and collaborative approach is essential in navigating the challenges posed by odontogenic keratocysts and ensuring optimal patient care and treatment efficacy.

## References

[1] Walsh M, Hussein MA, Carter M, Abdulrahman S (2022). Maxillary odontogenic keratocyst. J Surg Case Rep.

[2] MacDonald-Jankowski DS (2011). Keratocystic odontogenic tumour: systematic review. Dentomaxillofac Radiol.

[3] El-Naggar AK, Chan JK, Takata T, Grandis JR, Slootweg PJ (2017). The fourth edition of the head and neck World Health Organization blue book: editors' perspectives. Hum Pathol.

[4] Lund V (1985). Odontogenic keratocyst of the maxilla: a case report. Br J Oral Maxillofac Surg.

[5] Pylkkö J, Willberg J, Suominen A, Laine HK, Rautava J (2023). Appearance and recurrence of odontogenic keratocysts. Clin Exp Dent Res.

[6] Mohamed AA, Babiker AA, Khalfallah MS, Eltohami YI (2023). Odontogenic keratocysts: presentation and surgical outcome in a sample of sudanese patients. Int J Dent.

[7] Chirapathomsakul D, Sastravaha P, Jansisyanont P (2006). A review of odontogenic keratocysts and the behavior of recurrences. Oral Surg Oral Med Oral Pathol Oral Radiol Endod.

[8] Urs AB, Kumar P, Singh S, Mohanty S, Chaudhary Z (2024). Odontogenic keratocysts: a retrospective histopathological study. Natl J Maxillofac Surg.

[9] Stoelinga PJ (2005). The treatment of odontogenic keratocysts by excision of the overlying, attached mucosa, enucleation, and treatment of the bony defect with carnoy solution. J Oral Maxillofac Surg.

[10] Voorsmit RA (1985). The incredible keratocyst: a new approach to treatment. Dtsch Zahnarztl Z.

[11] Dewan H, Patel H, Pandya H, Bhavsar B, Shah U, Singh S (2022). Mucormycosis of jaws - literature review and current treatment protocols. Natl J Maxillofac Surg.

[12] Ferguson B (2000). Fungus balls of the paranasal sinuses. Otolaryngol Clin North Am.

[13] Mahjoub SBM (2024). Maxillary odontogenic keratocyst suprimposed by infection: case report. J Clin Med Surgery.

[14] Sheethal HS, Rao K, H S U, Chauhan K (2019). Odontogenic keratocyst arising in the maxillary sinus: a rare case report. J Oral Maxillofac Pathol.

[15] Palem SR, Devarakonda V, Navakoti P, Pendyala KS (2023). A rare case of odontogenic keratocyst in the maxillary sinus: diagnosis and management. Malawi Med J.

[16] Singh S, Shukla P, Bedi RS, Gupta S, Acharya S (2023). An unusual case of maxillary sinus odontogenic keratocyst: an insightful report with review of the literature. Cureus.

[17] More C B, Saha N (2016). Maxillary sinus polyp: an analysis on computed tomography. IP Int J Maxillofac Imaging.

[18] Mighic A, Sîrbu D, Mostovei A, Eni S (2024). Fungal sinusitis of the maxillary sinus. Medicina Stomatologică.

[19] Essaket S, Benjelloun L, Chbicheb S (2021). Odontogenic keratocyst mimicking a radicular cyst. Integr J Med Esci.

[20] Farooq S, Kak M, Shah A (2022). Management of odontogenic keratocysts using topical 5-fluorouracil: a comparative study. J Pharm Negat Results.

[21] Widyandhika CA, Anggraini JA, Yusuf HY (2023). Potential recurrence of odontogenic keratocyst post-surgery. J Syiah Kuala Dent Soc.

